# An Exposure-Based Video Game (Dr. Zoo) to Reduce Needle Phobia in Children Aged 3 to 6 Years: Development and Mixed Methods Pilot Study

**DOI:** 10.2196/42025

**Published:** 2023-10-16

**Authors:** Pat Healy, Celine Lu, Jennifer S Silk, Oliver Lindhiem, Reagan Harper, Abhishek Viswanathan, Dmitriy Babichenko

**Affiliations:** 1 University of Pittsburgh School of Computing and Information Pittsburgh, PA United States; 2 University of Washington Department of Psychology Seattle, WA United States; 3 University of Pittsburgh Department of Psychology Pittsburgh, PA United States; 4 University of Pittsburgh School of Medicine Department of Psychiatry Pittsburgh, PA United States; 5 Duquesne University Gumberg Library Pittsburgh, PA United States

**Keywords:** needle phobia, serious games, children, exposure therapy, cognitive behavioral therapy, anxiety, mobile phone

## Abstract

**Background:**

Needle phobia, which affects 19% of children aged 4 to 6 years, prevents many children from receiving necessary or preventive medical treatments. Digital interventions have been made to target needle phobia but currently rely on distraction rather than evidence-based exposure.

**Objective:**

We designed and evaluated a serious exposure-based mobile game called Dr. Zoo to reduce the fear of needles in children aged 3 to 6 years, where players administered shots to cartoon animals.

**Methods:**

We conducted a mixed methods study with 30 parents (mean age 35.87, SD 4.39 years) and their 36 children (mean age 4.44, SD 1.11 years) who played the game for 5 days leading to a scheduled appointment that included an injection (eg, influenza vaccination). After the study, parents completed exit surveys and participated in semistructured interviews to evaluate ease of use, acceptability, and preliminary effectiveness of the game and to provide insights on their experience with the game to inform future developments. Interview transcripts were analyzed by 3 independent coders following an open coding process and subsequently coded and discussed to reach consensus.

**Results:**

Parents rated their child’s difficulty in completing the game as very low on average (scale 1-5; mean 1.76, SD 0.82) and were highly likely to recommend Dr. Zoo to other parents (scale 1-5; mean 4.41, SD 0.87), suggesting Dr. Zoo’s strong ease of use and high acceptability. In the exit survey, parents rated their child’s fear as significantly lower after participating in the study (scale 1-5; mean 3.09, SD 1.17) compared with that before participating (scale 1-5; mean 4.37, SD 0.81; *z* score=−4.638; *P*<.001). Furthermore, 74% (26/35) of the parents reported that the game had a positive impact on their child’s fear or perception of needles (only 2 parents reported a negative impact). Qualitative analysis of the interview transcripts revealed potentially important features of the game in this positive impact, such as the game’s interactive design, as observed in 69% (24/35) of our participants.

**Conclusions:**

The results suggest that an evidence-based serious mobile game can be an easy-to-use, acceptable, and potentially effective intervention for changing young children’s fear and perceptions of needles. Leveraging digital interventions may be a potential solution to needle anxiety as a public health concern.

## Introduction

### Background

The fear of needles is a critical health barrier for millions of children worldwide, with approximately 19% of children aged 4 to 6 years experiencing needle or injection phobia [1]. Needle phobia in children is also associated with distress and avoidance of medical care for chronic pediatric conditions that require routine injections, such as cystic fibrosis [2] and diabetes [3]. Needle phobia typically begins in childhood, and if left untreated, can follow into adulthood [4], which can result in the reduced uptake of vaccines, such as the influenza vaccine [5] and COVID-19 vaccine [6], as well as avoidance of other routine procedures such as blood tests, pain relief measures, and blood donation in adults [7]. Therefore, there is a pressing public health need for acceptable, scalable, accessible, and lasting approaches to treating needle phobia in children.

Although cognitive behavioral therapies (CBTs) are considered highly effective in treating needle phobias [8-11], it is neither cost-effective nor feasible to engage every patient with a mental health professional to undergo CBT. In recent years, video games and other game-like digital interventions that leverage CBT, such as virtual reality exposure therapy (VRET), have been shown to be effective in treating a variety of anxiety disorders [12,13], but no digital interventions using CBT have specifically focused on needle anxiety. Designing a similar intervention to reduce needle anxiety in young children offers numerous advantages, including scalability, cost, reduced number of office visits, and entertainment value [14-16].

Our goal is to leverage these benefits of digital intervention to improve the design and to evaluate the ease of use, acceptability, and preliminary effectiveness of an engaging and scalable digital game called “Dr. Zoo” to reduce the fear of needles and injections in children aged 3 to 6 years. We have developed a pilot version of the game, which presents children with scenarios in which the players must deliver a needle injection to a sick animal to make it feel better. We hypothesized that through repeated exposure to the in-game needle, the players (ie, children) will become more comfortable with needles and shots, which in turn will reduce needle-related anxiety. We have pilot-tested a preliminary mobile version of this game with children and their families in the Greater Pittsburgh region. The goal of this paper is to describe the development process of Dr. Zoo, including the rationale for the design, game mechanics, and presentation modality decisions. Furthermore, we report the preliminary results of several user studies and a pilot feasibility study with 36 children.

### Related Works

In recent years, video games using CBT approaches have been developed with the purpose of combating anxiety as an inexpensive alternative to more conventional therapeutic methods. For example, Carlier et al [17] created a video game (New Horizon) to reduce anxiety in children with autism through relaxation techniques pulled from CBT [17], and Heng [18] created ReWIND, a role-playing video game that embeds an antecedent-belief consequence model from CBT into gameplay to treat patients with generalized anxiety disorder [18]. In these 2 examples, we see support for the application of CBT strategies in serious games that target anxiety in children.

For our problem domain of needle anxiety in children, virtual reality (VR) games that distract patients during the injection process have recently become a popular intervention, although there is little support for their long-term effects on anxiety reduction. A systematic review and meta-analysis of 10 randomized controlled trials found that overall, VR as a distraction significantly reduced children’s fear of needles while undergoing needle procedures compared with children who did not receive the VR distraction [19]. However, there is no empirical evidence supporting the effectiveness of distraction-based games in producing long-term changes in children’s fear of needles beyond the immediate experience itself. This suggests that a child who fears needles would need to undergo the distraction intervention for each future needle procedure because their underlying anxiety is not being resolved.

Our study departs from this distraction approach, instead focusing on exposure, which has empirical support for decreasing anxiety in the long term. Unlike distraction, exposures aim to modify a child’s perception of needles to reduce fear in the long term rather than merely providing an in-the-moment solution. Exposure is a core element of CBT for child anxiety, which has been shown to be particularly effective in treating needle phobia [20]. Exposure involves presenting a child with their feared stimulus, in this case a needle, in repeated trials until the child no longer has a fear response to the stimulus. Furthermore, a gradual exposure approach where a less-feared version of the stimulus is presented first (eg, a needle in a video game) before the actual feared stimulus (eg, an actual needle during a medical appointment) can make treatment more palatable to the patient and prevent treatment dropout [21]. Through habituation to the feared stimulus (ie, a needle), a child’s fear of the stimulus decreases and they learn that they can manage the situation. Therefore, they no longer need to engage in behaviors to avoid the feared stimulus (ie, a needle) to see a decrease in fear. A cognitive shift follows exposures, altering a child’s perceived threat of the stimulus and their ability to cope with the threat [21,22].

Although there is no established literature on games that leverage exposure to tackle needle anxiety, there is substantial prior work in VRET games that leverage exposure to combat other forms of anxiety. Our study does not concern itself with VR directly, because we developed a *normal* or *pancake* game (as in a video game that is not VR), but this body of work in VR is necessary to discuss because it is the typical modality of digital interventions that leverage exposure. As discussed by Walkom [23], VRET is a uniquely effective and safe form of exposure therapy because of the precise control the designer has on the appearance and intensity of the stimulus and the ability of the participant to quickly abandon the simulation if the stimulus becomes overwhelming [23]. A review of 23 studies comparing VRET approaches to classical face-to-face evidence-based treatments in treating anxiety disorders found that VRET was superior to the waitlist control and comparable to traditional methods in terms of efficacy, impact, and stability of results [11].

However, little is known about the effect of less-immersive, exposure-based games (ie, non-VR) on anxiety. Exposure-based serious games that are neither VR games nor augmented reality games are exceptionally rare. In our review of the literature, we found only 1 example: a game named Lumi Nova, which was developed to address a wide range of anxiety issues (generalized anxiety, separation anxiety, social anxiety, panic disorder, and agoraphobia) in children aged 7 to 12 years [12]. Given its broader approach to helping children address a wider range of anxieties, this game does not primarily focus on exposing players to visual stimuli that resemble the object of their anxieties, as in the VRET examples discussed, but instead delivers a narrative where the player engages in puzzles to help in-game characters overcome their anxieties and facilitate goal-setting for exposure activities the player engages in outside the game [12]. In a user study of Lumi Nova (n=30), Lockwood et al [12] found that the game led children to a small but statistically significant decrease in overall anxiety after 8 weeks of play, as assessed by the player’s parent or guardian using the Spence Child Anxiety Scale [12].

Our game, Dr. Zoo, has a “flat” modality, which lends itself to two core advantages over VRET: (1) it is less expensive and (2) it is more scalable, not requiring any special VR hardware outside of a mobile device. The strengths of VRET over conventional therapies may also apply to the “flat” modality of conventional mobile games, such as the designer’s ability to precisely control the intensity and duration of the stimulus, the safety that comes with the player’s ability to abandon the game at any point if the stimulus becomes overwhelming, and the added dimension of empathetic experiences between the patient and nonplayer characters. The impact of “flat” exposure-based games on anxiety in clinical settings will be further explored in this study. Furthermore, unlike the distraction methodology that provides only a short-term remedy in serious games for needle anxiety [24], we opted for an exposure therapy approach to create a more sustained effect by altering patients’ responses to, perceptions of, and ability to cope with needles.

### Objectives

In this paper, we present (1) insights into the design of a digital game to change perceptions of needles and injections in children aged 3 to 6 years and (2) insights into the ease of use, acceptability, and preliminary effectiveness of a serious game based on feedback from the players’ parents.

## Methods

### Design Approach

We designed Dr. Zoo, a 3D adventure game for mobile devices (tablets and smartphones), as a single-player serious game to assist children in reducing their fear of needles. Above all else, our goal was exposure therapy: we wanted to create a game that requires its players to observe and interact with needles. Therefore, we decided that the game should focus on repeating a scenario in which the player must administer a vaccine to a patient. This scenario would be free of distraction, with only minimal visual elements outside of the syringe and patient, compelling the player to directly engage with the syringe for the duration of the scene. After multiple informal unstructured interviews with parents and children in our prospective age group, we decided to represent in-game virtual patients as cartoon animals. On the basis of which animals were popular among these children and which 3D animated assets we had access to, we decided that the game should center around a dragon, a dog, a penguin, a polar bear, and an orca.

We anticipated that the game would be more successful in addressing needle anxiety if the player empathized with their “patients.” Our goal was to show animals in distress, have the player comfort these animals to calm them down before injecting a medication, administer the injection, and see a positive effect of the injection.

We had originally conceptualized the game as a series of injection-focused scenarios, which meant a constant presence of needles in every scene. However, in early playtests, we noticed that if the needle was always on the screen and the player was coming into the experience with especially high needle anxiety, the presence of a needle prevented players from becoming emotionally invested in interactions with the animals. This insight led us to a design decision to alternate needle and nonneedle scenes. For each animal, the player first engages in an animal-specific minigame with no needles present to build a connection and empathy between the player and the animal; these scenes do not necessarily contain therapeutically relevant content directly, instead they focus primarily on building their relationship with the character. Once the player has familiarized themselves with the animal, the scene changes to a scenario in which the animal becomes sick, and the player must administer an injection to make the animal feel better.

### Dr. Zoo

**Figure 1:**
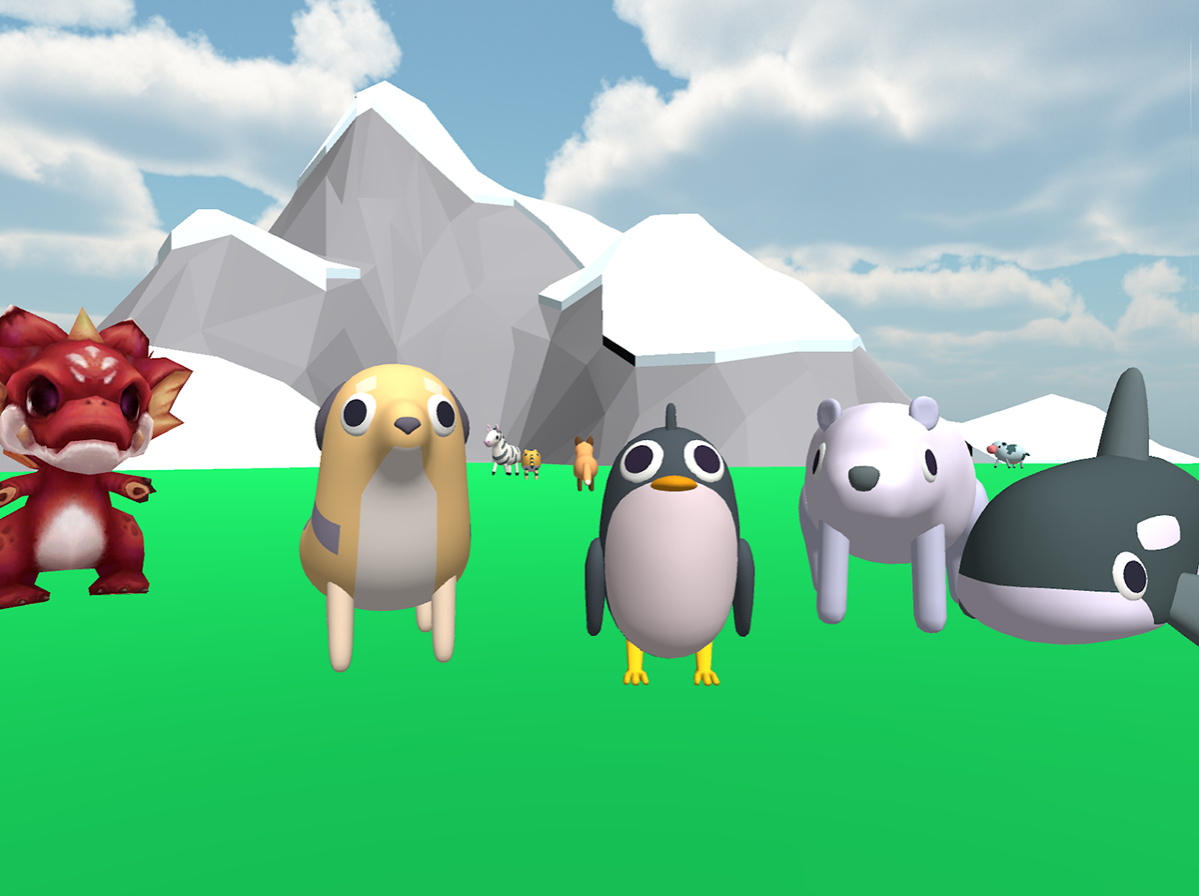
The character-select screen of Dr. Zoo. This is the screen where players choose which animal they will help next.

Dr. Zoo is a 3D adventure game we developed in Unity for mobile devices (Android and iOS smartphones and tablets). In a series of independent chapters, the player is introduced to an animal, plays a brief minigame to solve some problem that this animal is facing, and concludes the interaction by administering an injection to the animal. The version of the game deployed for the pilot study includes 4 chapters. Although the chapters were presented to players in a specific order (by animal; Figure 1), the players were free to select and play chapters in any order they liked.

**Figure 2:**
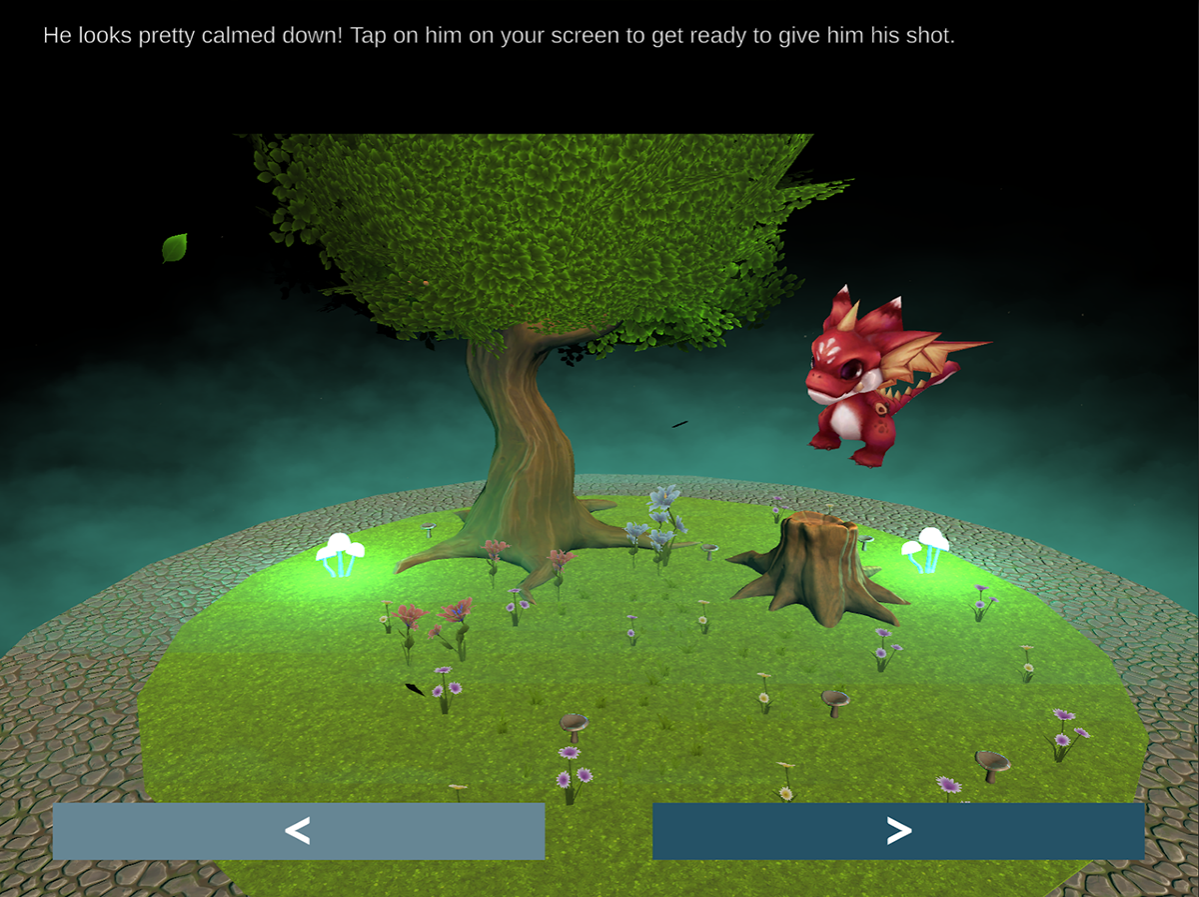
The end of the Dominic the dragon chapter.

**Figure 3:**
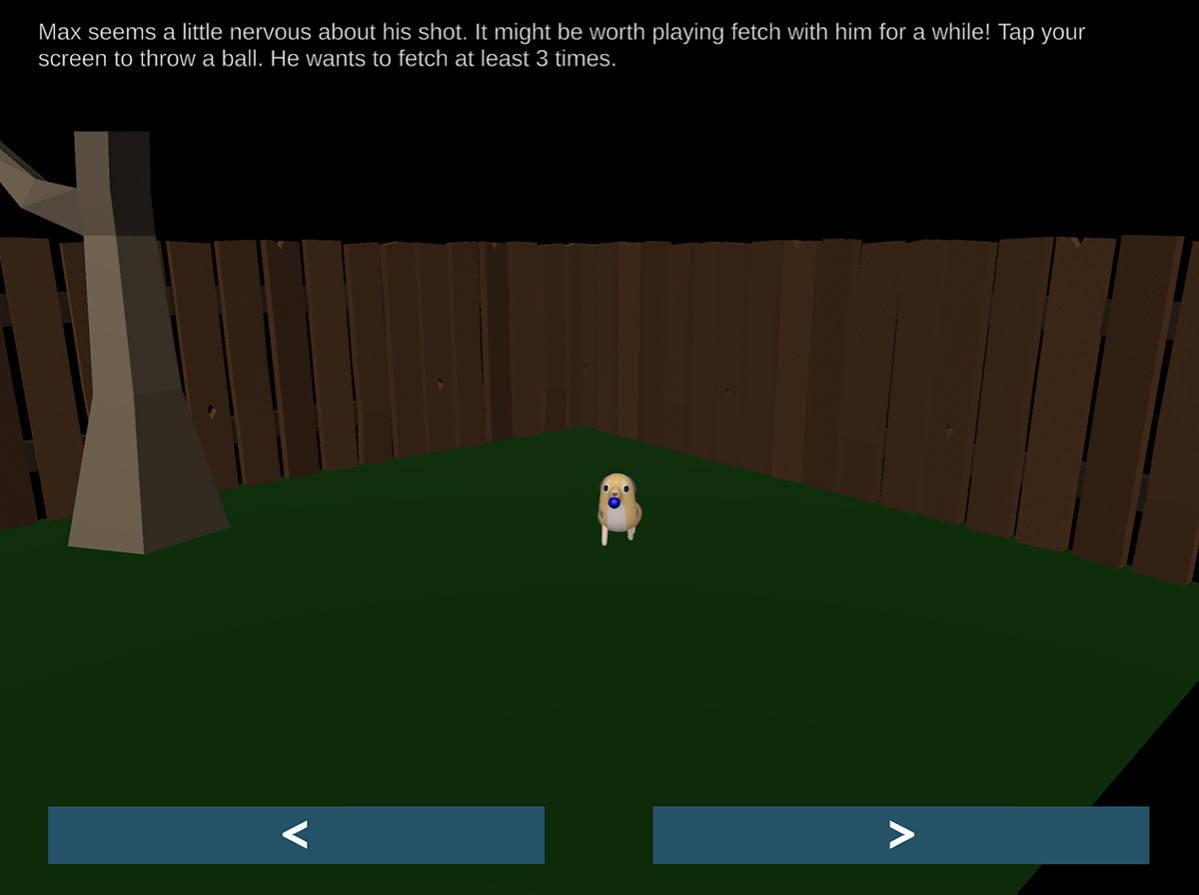
The player must play fetch with Max the dog in the second chapter.

The first 2 chapters present players with a patient (a dragon named Dominic, shown in Figure 2, and a dog named Max, shown in Figure 3) who is experiencing anxiety about their scheduled vaccination. In the first chapter, Dominic the dragon is anxiously flying around the game environment, and the player must use the microphone on their device to gently talk to him until he calms down. In the second chapter, the player must play fetch with Max to help him calm down, tapping the screen to toss a ball in a backyard environment.

**Figure 4:**
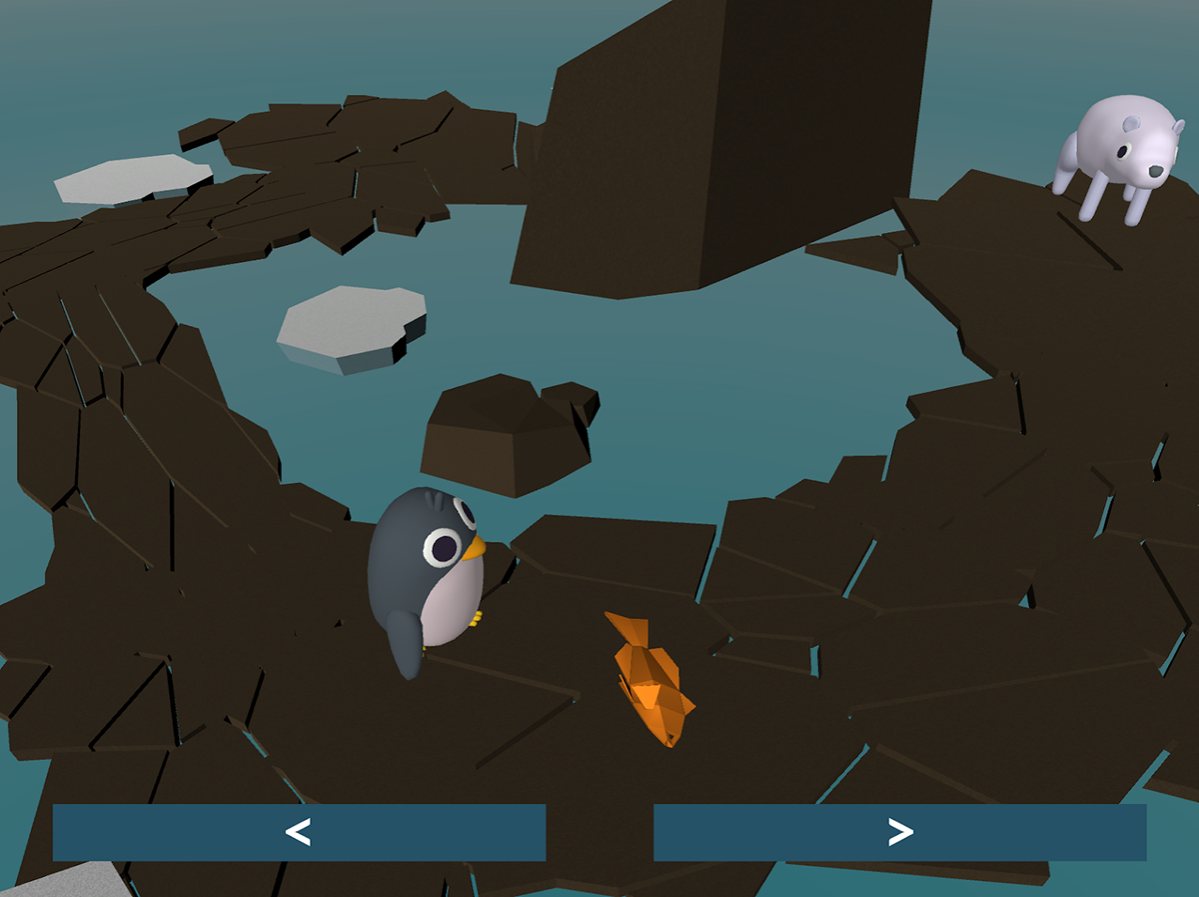
In the Penny the penguin chapter, the player must tap the screen to place the orange fish on the ground, which Penny will follow.

The third chapter features a penguin named Penny, who lost track of her friend, a polar bear named Becky. The player must guide Penny along a short path to find Becky. The player indirectly moves Penny by tapping the ground to place fish, which Penny will walk toward and eat, as shown in Figure 4. Upon guiding Penny to Becky, Becky falls ill and requires an injection.

**Figure 5:**
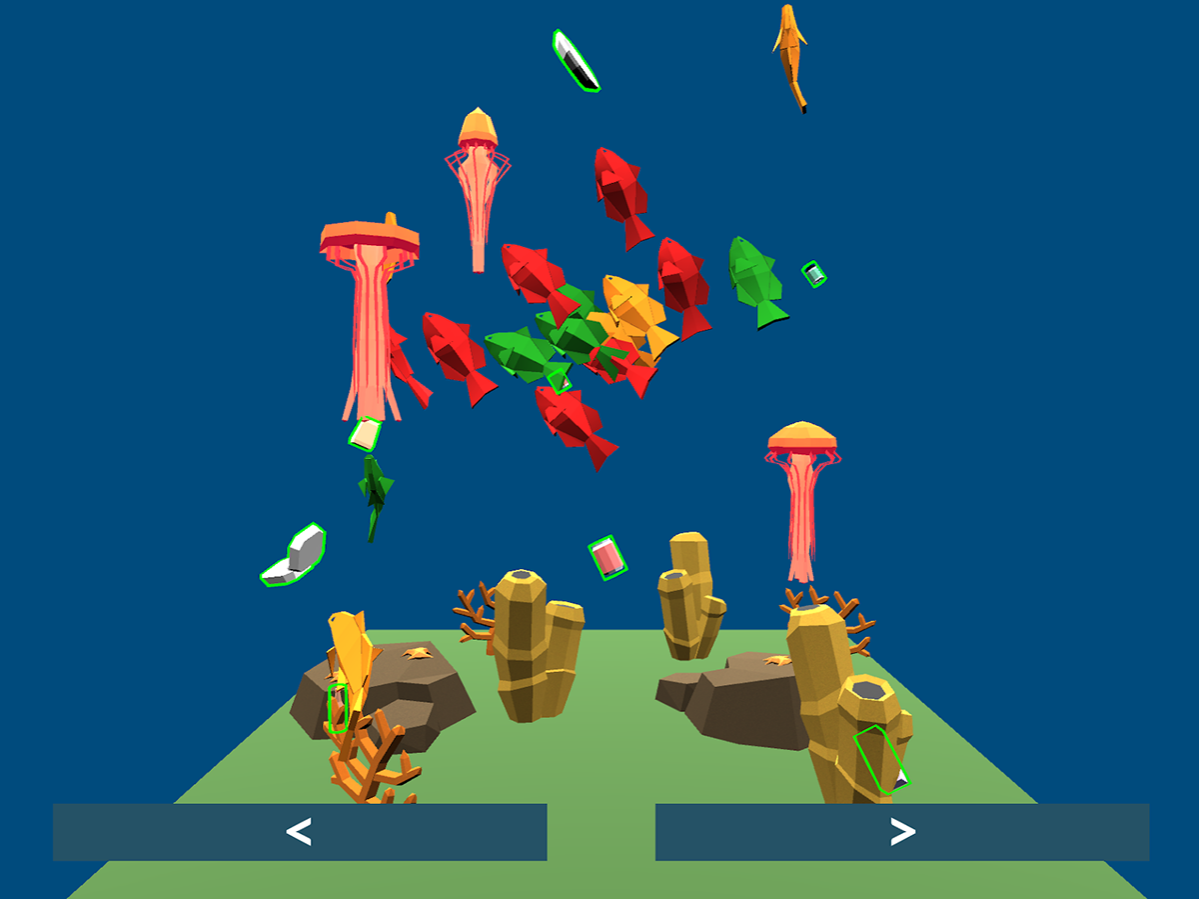
In the Sami the orca chapter, the player must search through the small ocean environment to tap on trash objects.

The fourth and final chapter features an orca named Sami, who cannot come to her appointment because her part of the ocean is full of trash. The player must search the game environment for pieces of trash among fish and other wildlife and tap on them to pick them up until all the trash has been collected, as shown in Figure 5.

The diverse set of animal-specific minigames that make up most of the content in all 4 chapters of Dr. Zoo was designed to offer an opportunity to grow fondness for the animal characters by having the player help them solve “health” problems with slightly different empathetic mechanisms. With Dominic the dragon, the player must engage verbally with the character to assist them in overcoming their anxiety. With Max the dog, the player must engage in open-ended play with a physical object (the ball), which looks to put both the player and, in the fiction of the game, the dog together in a flow state. With Becky the polar bear, the player’s emotional connection is cultivated with a design strategy unique to games: searching for the character is a gameplay goal. Finally, Sami the orca’s chapter takes a similar approach, pushing the player to empathize with the character through a simplified appeal to environmentalism, such that Sami only makes her triumphantly animated entrance after the player has cleaned up ocean trash. Therefore, we have 4 distinct modes of engagement with our animal patients: verbal conversation, flow-inspiring action, searching, and service.

This progression in the modes of engagement mirrors the progression in the player’s relationship with the animal character, because the player experiences a wide range of character interactions from direct emotional support as if the player is a caregiver (dragon) to incidental emotional support through play (dog), to indirect care through a friend of the character (controlling the penguin to reach the polar bear), and to action that appears to have only mild relation to the character (orca). Overall, Dr. Zoo presents a useful spectrum of player and nonplayer character interactions in this context, allowing both an exploratory case study to further understand, through our feasibility study, which kinds of interactions may resonate most often in this context and, assuming players may individually differ in which interactions they prefer, crafting an experience where most players enjoy at least one of the nonplayer character interactions, even if that choice is not universal.

Our design was motivated by the idea that once a fondness for a given character is established, the player will become more invested in the impact of the injection on the animal’s well-being, therefore encouraging the player to role-play as an advocate for the injection, both for their patient and, by extension, themselves. This hypothesized mechanism resembles prior work in the world of “identification,” in which players self-identify with the characteristics of nonplayer characters [25]. In our game, we may see players self-identify with the courage of our virtual patients in the face of injections.

In addition, this mechanism is supported by social learning theory, which conceptualizes social behaviors as learned in part through observation and imitation [26]. In the case of Dominic the dragon’s chapter, for example, the player is observing the nonplayer character’s changing behavior as it manages its anxiety, so that the player may imitate that reduction of anxiety in their own conduct.

**Figure 6:**
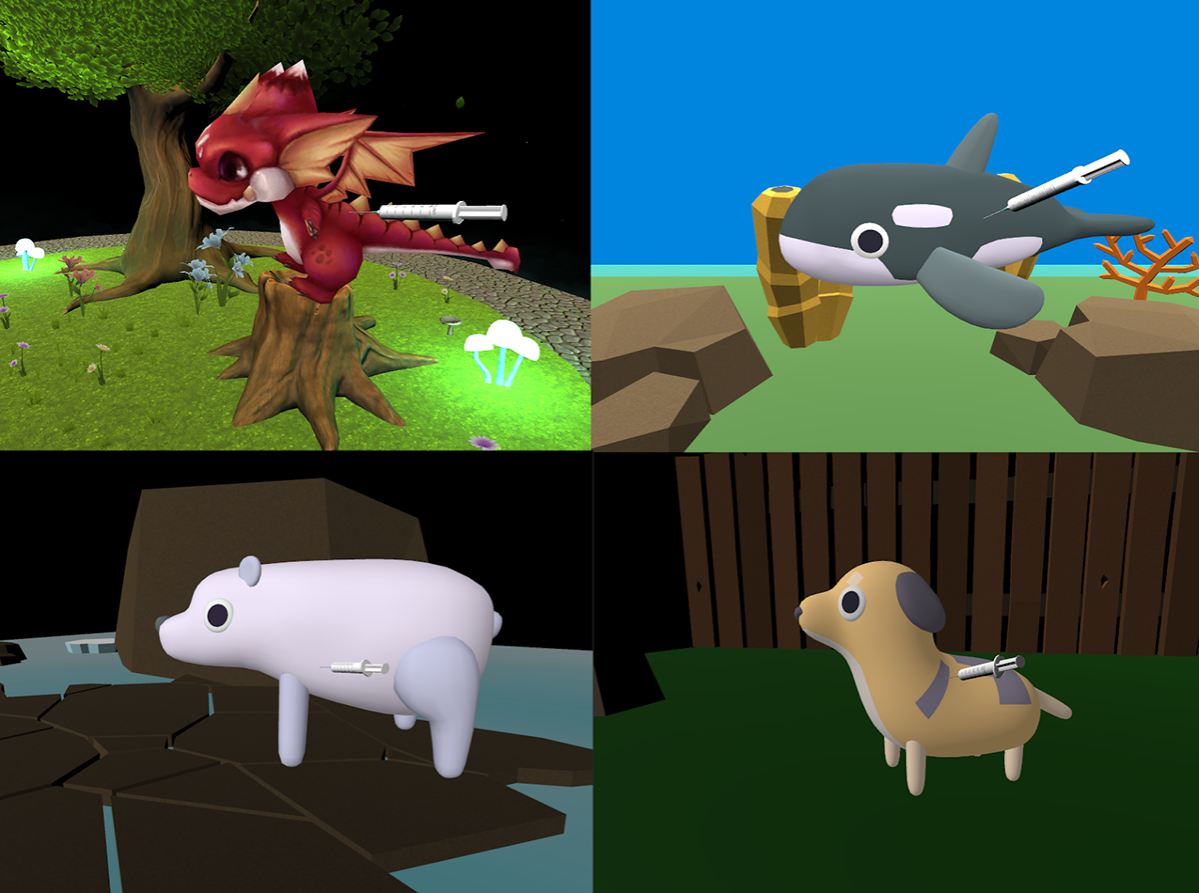
Characters in Dr. Zoo receiving an injection administered by the player.

**Figure 7:**
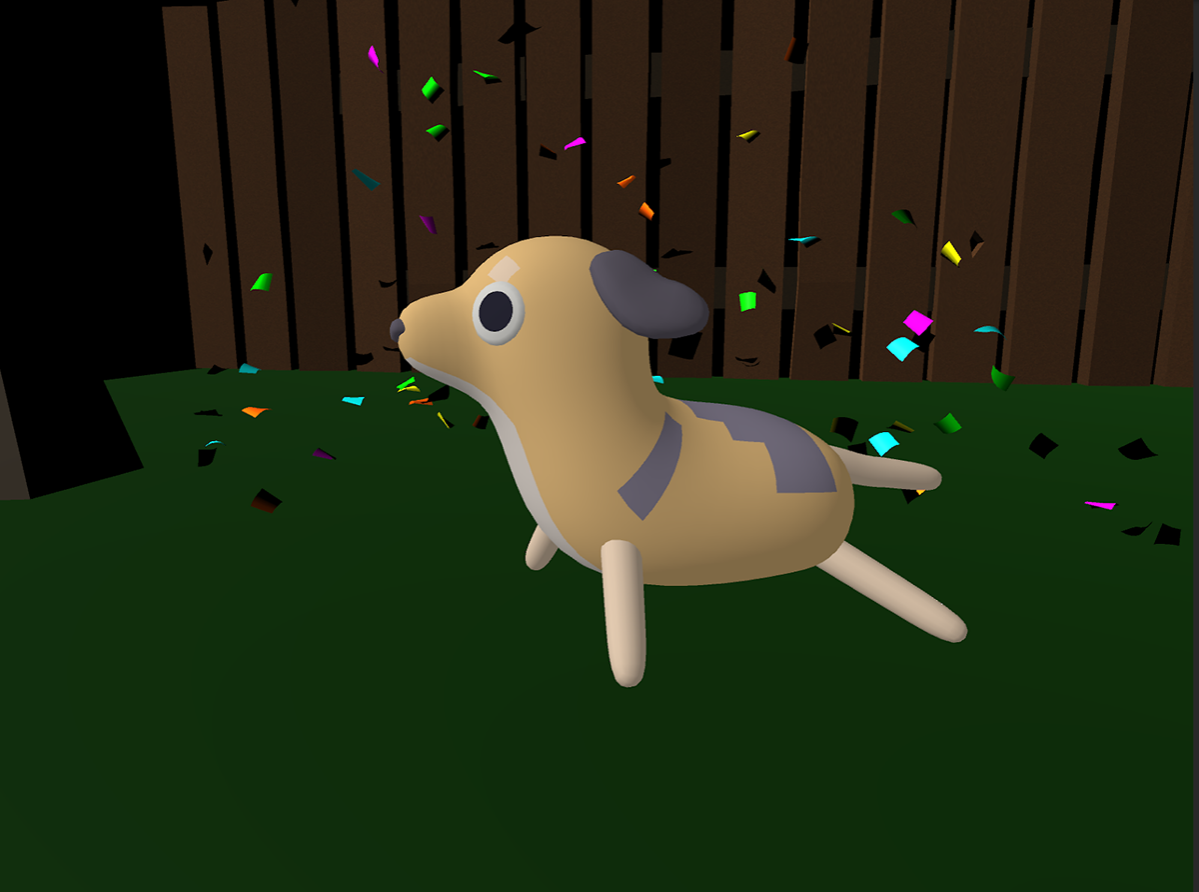
A celebration sequence that occurs after the player has administered an injection.

Each chapter concludes with the player administering an injection to their animal patient, as shown in Figure 6. In these scenes, the player must tap and drag a syringe into the animal’s arm and hold it there for about 3.5 seconds. As the syringe is held in this position, a circular loading icon is filled, indicating progress. When the circle is filled, the syringe is removed from the screen and the patient becomes visibly more energized and does a celebratory dance with an audible cheering sound effect and confetti, as shown in Figure 7. From social learning theory, we can understand this celebration as a kind of vicarious reinforcement [26], as the player has watched the nonplayer character accept the injection and be rewarded with a celebration for being a good patient. Upon completing 1 chapter, the player is returned to a chapter-select screen to choose their next patient. The player cannot replay any chapters before completing all the 4 chapters, which triggers a finale sequence where all the animals celebrate the player’s success and the game automatically restarts itself.

### Research Design

The pilot feasibility study followed a within-subjects design and compared parent report of child participants’ fear ratings after the initial game session and final game session. The study also compared parent retrospective reports of their child’s fear of needles and distress (eg, screaming, kicking, and crying) during needle-related activities in the past years before participating in the study and parent reports of their child’s fear and distress during needle-related activities after participating in the study. Qualitative exit surveys were conducted to gain insights into the ease of use, acceptability, and preliminary effectiveness of the game. Furthermore, qualitative interviews were conducted to gain insight into parents’ and children’s experiences with the game, to inform changes for future development.

### Participants

Participants were 36 children aged 3 to 6 years (mean age 4.44, SD 1.11 years) and 30 parents (mean age 35.87, SD 4.39 years). A total of 6 parents in the study enrolled 2 children to participate. Children were considered eligible for participation if they (1) were aged between 3 and 6 years; (2) previously experienced an injection, intravenous therapy, or any other activities involving syringes or needles as part of a medical treatment; (3) experienced needle anxiety based on parent report; and (4) had an upcoming medical appointment that involved syringes or needles (eg, influenza vaccination).

Children were excluded from participation if they either (1) had a fear of animals that were present in the game (ie, orca whales, penguins, polar bears, dogs, or dragons) or (2) had any seizure disorder, because some scientific literature suggests that video games could trigger seizures [27]. The demographic information of the participants is presented in Tables 1 and 2.

**Table 1:** Overview of participant demographics (children; n=36).

Variables		Values, n (%)
**Sex**		
	Female	22 (61)
	Male	14 (39)
**Race**		
	White	28 (78)
	White and Asian	2 (6)
	White and Black or African American	1 (3)
	Biracial other	3 (8)
	Prefer not to answer	2 (6)
**Ethnicity**		
	Non-Hispanic	28 (78)
	Hispanic	8 (22)

**Table 2:** Overview of participant demographics (parents; n=30).

Variables		Values, n (%)
**Sex**		
	Female	29 (97)
	Male	1 (3)
**Race**		
	Black or African American	1 (3)
	White	26 (87)
	White and Asian	1 (3)
	White and Black or African American	1 (3)
**Ethnicity**		
	Non-Hispanic	28 (93)
	Hispanic	2 (7)
**Income (US $)**		
	<24,999	1 (3)
	25,000-49,999	2 (7)
	50,000-74,999	4 (13)
	75,000-99,999	3 (10)
	>100,000	20 (67)

### Ethics Approval

All research procedures were approved by the University of Pittsburgh's Institutional Review Board (STUDY20090225).

### Procedures

#### Recruitment and Screening

Participants were recruited between May and November 2021 through a web-based university-sponsored research registry for families interested in participating in behavioral health research studies. Participants who indicated an interest in the study through the research registry were sent a prescreening Qualtrics (Qualtrics International Inc) survey to complete. Screening was performed in 2 phases. In phase 1, if the participants indicated that their child met the inclusion criteria, the research staff contacted the potential participant to schedule a web-based consent visit. A research assistant conducted the virtual consent visit either through phone or video call through Microsoft Teams, a videoconferencing platform, explaining the purpose of the study, overall procedures of the study, and risks and benefits of participation and answering any questions the parent had about the study. Parent participants then completed a web-based consent form via Qualtrics. In phase 2 of screening, following consent, participants were sent baseline surveys to complete, including a demographics survey and the Fear Survey Schedule for Children–Revised (FSSC-R) [28]. Final eligibility criteria were met based on parents reporting that their child had at least “some” fear on the FSSC-R needle phobia item.

#### Gameplay

Approximately 2 weeks before the scheduled medical appointment involving needles or syringes, research staff instructed parents on how to download Dr. Zoo onto their smartphone or tablet device. Dr. Zoo is compatible with both Android and iOS devices. Given its low-polygon style and limited action, it does not require new or powerful hardware to run (it was tested with acceptable performance on an inexpensive Android tablet during development). Participants using an Android device were required to download and install an Android Package Kit file manually, whereas iOS users received the app through Apple’s TestFlight platform. Parents were instructed to have their child complete all 4 chapters of Dr. Zoo each day for at least 5 days in a row, leading to their child’s medical appointment. When their child finished playing for the day, parents completed the Children’s Fear Scale [29] via a Qualtrics link embedded into the game to indicate their child’s fear level while playing Dr. Zoo. No other instructions were given to parents on how to determine their child’s fear level during the game. Parents also received daily SMS text message reminders to have their child play the game and complete the survey during their scheduled gameplay period.

#### Qualitative Exit Interview

**Figure 8:**
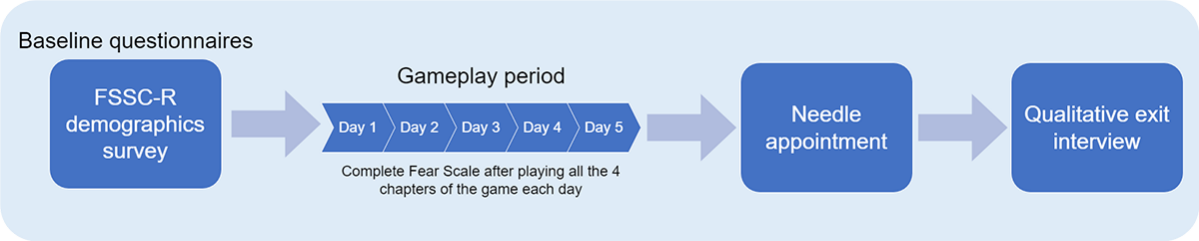
A flowchart depicting the timeline of assessments in the study. FSSC-R: Fear Survey Schedule for Children–Revised.

Upon completing the medical appointment, the research staff scheduled and later completed an exit survey and interview using a semistructured interview guide with the parents (refer to Figure 8 for the timeline of assessments). Exit interviews were conducted and audio recorded on Microsoft Teams, with all but one parent (final sample of parent interviews, n=29) because the research staff were no longer able to reach the parent. The interviews lasted between 9.73 and 30.60 (mean 17.53, SD 5.38) minutes. For parents who enrolled 2 children in the study, the exit interview was conducted once with the parent while probing experiences for each child separately. The Microsoft Teams application autogenerated transcripts of the interviews, which research assistants later checked and revised manually for accuracy. Families were compensated US $20 for each child that participated in the study following the completion of the exit interview.

#### Protection of Health Information

Participant health information was protected using several methods. All data were stored in the university-managed OneDrive (Microsoft) database, which required research staff to log in using their university credentials to gain access. Participant data were tracked using password-protected Microsoft Excel sheets that only approved research staff had access to. Participant data were kept separate from their identifiable information, such as name, date of birth, or contact information. Furthermore, research assistants deidentified and then saved the qualitative exit interview transcripts by removing information such as name or date of birth.

### Instruments and Measures

#### The Fear Survey Schedule for Children–Revised

The FSSC-R [28] was used to determine eligibility for the study. The measure is a widely used questionnaire that measures the number of fears and the overall level of fearfulness in children. The item used to determine eligibility for this study assesses children’s fear of “Getting a shot from the nurse or doctor,” on a 3-point Likert scale (0=“none,” 1=“some,” and 2=“a lot”). Parents had to report that their child had at least “some” fear to be eligible for participation.

#### Demographics

Parents reported on demographic variables such as parent and child age, sex, race, ethnicity, and household income.

#### Children’s Fear Scale

Parents completed the Children’s Fear Scale [29] to measure children’s fear levels while playing the game. This measure is a 5-point visual scale that presents 5 human faces with expressions showing different fear intensities (0: leftmost face=“not scared at all” to 4: rightmost face=“most scared possible”). Parents were asked to choose the face that best represented their child’s response while playing Dr. Zoo. The Children’s Fear Scale was used to measure the child’s fear while playing the game each day.

#### Exit Interview

The exit interview consisted of a semistructured interview guide that asked participants about a combination of scaled questions (ie, exit survey) and open-ended questions. This allowed participants to elaborate and contextualize their responses to the scaled questions. The semistructured interview guide was developed with specific guiding questions to elicit feedback on (1) parents’ and children’s favorite aspects of the game, (2) parents’ and children’s least favorite aspects of the game, (3) parents’ perceived impact of the game on their child’s fear of needles or distress, and (4) suggestions for future development. A semistructured interview guide consists of guiding questions and suggested probing questions to evoke more details from the participant, if needed. Questions can be asked in different orders to match the flow of the conversation based on the participants’ responses.

To assess ease of use, we asked parents to rate how difficult it was for their child to understand how to play the game on a 5-point Likert sale (1=“extremely easy” to 5=“extremely difficult”). To assess acceptability, parents were asked how likely they were to recommend the game to other parents on a 5-point Likert scale (1=“absolutely not” to 5=“definitely yes”) as well as how helpful they thought the game was in reducing their child’s fear of needles on a 5-point Likert scale (1=“did not help at all” to 5=“helped a lot”).

Finally, to assess preliminary effectiveness, parents were also asked to retrospectively rate their child’s fear of needles during needle-related activities in the years before participating in the study and after participating in the study on a 5-point Likert scale (1=“no fear at all” to 5=“extreme fear”). Similarly, parents were asked to retrospectively rate their child’s distress during needle-related activities (eg, kicking, screaming, and crying) in past years before participating in the study and after participating in the study on a 5-point Likert scale (1=“completely calm” to 5=“extremely agitated”).

### Data Collection and Analysis

Baseline and fear scale surveys were administered using Qualtrics.

#### Coding

A total of 3 coders—one of the first authors (postbaccalaureate) and 2 collaborators (1 master’s student and 1 PhD graduate student)—were involved in the qualitative analysis process. To guide our qualitative coding process, we used a combination of deductive and inductive coding. As part of a deductive coding approach, we used the framework of the interview script to develop four distinct categories that potential codes could fall under: (1) facilitators of use, (2) barriers to use, (3) impact of the game, and (4) suggestions for future development. Then, coders used an open coding process, as described by Strauss and Corbin [30], and independently reviewed the same 6 transcripts, identifying important or repeated concepts in the transcripts, while placing them into 1 of the 4 categories. An initial codebook was then developed and used on 3 other transcripts, wherein each coder coded 1 transcript. The 3 coders met to discuss any concepts that the initial codebook failed to capture and revised the codebook to include new codes and changes to code definitions accordingly.

In the next phase, the 2 collaborators independently coded all transcripts using the revised codebook. Each transcript was coded by 2 coders, and the first coder of each transcript was responsible for segmenting the transcript into excerpts to facilitate the coding process. The coders were instructed to apply only 1 code to each excerpt. After all transcripts were double-coded, the 3 coders met to engage in an open discussion [31] to resolve any discrepancies between codes. Each coder explained their rationale for applying a specific code to an excerpt while the others listened. A final code was determined once the group reached a consensus on which code best suited the excerpt after hearing each other’s rationale. Changes to the codebook were made as necessary to capture the codes better. All coding was performed using Microsoft Word and Excel.

#### Quantitative Analysis and Triangulation

Quantitative data were analyzed using SPSS 28 (IBM Corp). Logistic regressions were conducted to identify the relationships between participants’ age and endorsement of certain qualitative codes. Triangulation of the data sources strengthened the validity of our findings and provided a more comprehensive depiction of participants’ experiences with the game.

## Results

### Overview

In the following section, we describe quantitative, qualitative, and mixed methods findings that focus on the following aspects of the game: (1) facilitators of use (including ease of use and acceptability), (2) barriers to use, (3) impact of the game (ie, preliminary effectiveness), and (4) suggestions for future development.

### Missing Data

We were unable to contact 1 parent participant after they completed their medical appointment, who did not complete the exit interview. One parent participant did not complete the fear scale survey for their child during their scheduled gameplay period. Finally, 1 parent participant was not asked to rate the difficulty in understanding because of researcher error.

### Descriptive Statistics

The average scores for the FSSC-R needle phobia item were very high (scale 0-2; mean 1.89, SD 0.32), indicating a high level of baseline needle anxiety among the children in the sample. Participants completed the game on average for 5.11 (SD 1.21) days.

Transcripts were segmented into 360 excerpts in total. The codebook consisted of 17 codes, separated into 4 categories. Overall interrater reliability, as measured by Cohen κ [32], was high across all codes (pooled κ=0.86), ranging from moderate agreement to near-perfect agreement (0.59-0.98). Table 3 presents the frequency of codes, the percentage of participants who endorsed the code, and the respective κ statistic scores for each code.

**Table 3:** Frequency and κ statistic scores for qualitative codes by category.

Category and code			Frequency of code, n		Participants who endorsed code (n=35), n (%)		Cohen κ^a^
**Facilitators to use**							
	Ease of use	8		8 (23)		0.93	
	Excitement to play game	24		18 (51)		0.98	
	Dominic the Dragon	22		15 (42)		0.98	
	Interactive	42		24 (69)		0.92	
**Barriers to use**							
	Difficulty navigating space	25		18 (51)		0.94	
	Injection sequence	24		14 (40)		0.93	
	Loss of interest	14		10 (29)		0.85	
	Other barriers	5		4 (11)		0.59	
**Impact of the game**							
	Positive impact on fear	31		21 (60)		0.79	
	Positive impact on physical reactions	21		17 (49)		0.89	
	Discussing health impact of vaccines	24		21 (60)		0.81	
	Preparation for appointment	27		21 (60)		0.78	
	No impact	14		10 (29)		0.88	
	Negative impact	4		2 (6)		0.59	
**Suggestions for future development**							
	More realistic	40		25 (71)		0.98	
	More variety	23		18 (51)		0.94	
	Technical improvements	12		11 (31)		0.82	

^a^Cohen κ statistic score.

### Facilitators of Use

#### Quantitative Results

A summary of all quantitative findings is displayed in Tables 4 and 5. The ease of use was high, with parents rating the difficulty of their children’s understanding how to play the game as relatively low (scale 1-5; mean 1.76, SD 0.82). The acceptability of the game was relatively high, with parent participants being very likely to recommend Dr. Zoo to other parents (scale 1-5; mean 4.41, SD 0.87). In addition, parents rated the helpfulness of the game in reducing their child’s fear of needles as moderately high (scale 1-5; mean 3.49, SD 1.27).

**Table 4:** Summary of descriptive statistics.

Item	Values, mean (SD)	Values, median (range)	Values (n=36), n (%)
FSSC-R^a^ needle phobia item	1.89 (0.32)	2 (1)	36 (100)
Children’s Fear Scale ratings across all days	0.867 (1.24)	0 (4)	35 (97)
Game difficulty	1.76 (0.82)	2 (3)	34 (94)
Likeliness to recommend game to other parents	4.41 (0.87)	5 (3)	29 (97)
Helpfulness of game	3.49 (1.27)	3 (4)	35 (97)

^a^FSSC-R: Fear Survey Schedule for Children–Revised.

**Table 5:** Summary of Wilcoxon signed rank tests.

Variables	Median values before and after the study (range)	*z* score	*P* value
Children’s Fear Scale ratings	1-0 (4-4)	−1.624	.10
Fear ratings from the exit survey	5-3 (2-4)	−4.638	<.001
Distress ratings from the exit survey	5-3 (3-4)	−4.313	<.001

#### Qualitative Results

Parent participants reported many aspects of the game that they and their children found enjoyable. For example, 8 (23%) out of 35 children could “figure out what to do on their own” and that the game was “simple” for them to play. In addition, 18 (51%) out of 35 children were excited to play Dr. Zoo each day, either reminding their parents that they had not yet played the game for the day or even asking them to continue to play the game past the study play period.

Each of the chapters were favored by at least some of the parents and children, though the Dominic the dragon chapter was a clear standout, with 15 (43%) out of 35 children indicating that this chapter was their favorite aspect of the game. Parents felt that this chapter was the most helpful for their children and enjoyed hearing their children comforting the dragon by “mirroring” the phrases they would often use to calm down their children, such as the following:

It’s gonna be okay. This will help you stay safe. It’ll only hurt for a second.

Furthermore, 24 (69%) out of 35 children enjoyed that the game was interactive, for example, by being able to choose the first animal in the beginning or being the one to give the animal a vaccination. Another interactive aspect of the game that children particularly enjoyed was the celebration sequence that occurs after administering the injection to an animal, which includes confetti and characters in the game exclaiming “Yay!” Parents felt that this sequence helped their children create a positive association with vaccines.

### Barriers to Use

#### Quantitative Results

Though ease of use was rated overall as high as explained in the *Facilitators of Use* section, an ordinal regression revealed that an increase in age (expressed in years) was associated with a decrease in the odds of rating difficulty of understanding how to play the game as high, with an odds ratio of −0.794 (95% CI −1.464 to −0.124; Wald 𝜒^2^_1_=5.389; *P*=.02). In summary, parents of younger children reported that their child had more difficulty understanding the game compared with parents of older children.

#### Qualitative and Mixed Methods Findings

Parents reported aspects of the game that proved to be unengaging, frustrating, or unappealing for themselves or their children. Although logistic regressions revealed that age was not associated with reporting any 1 barrier code in particular (all *P* values >.05; refer to Multimedia Appendix 1 for details), 1 notable barrier reported by 18 (51%) out of 35 participants included struggling to navigate the spaces in the Penny the penguin and Sami the orca chapters. Parents reported that their children became frustrated when “they couldn’t figure out how to get to the polar bear, that you had to use those arrows to get around to see the polar bear.” In these instances, parents would often help their child with this sequence to continue with the rest of the chapter. Similarly, some of the children became frustrated during the Sami the orca chapter because the pieces of trash to pick up were “too tiny” and they “didn’t really get the whole rotating [the screen].” This issue was most prominent when families used a smartphone for the game rather than a tablet with a larger screen.

Another aspect of the game that 14 (40%) out of 35 participants had an issue with was the injection sequence. Parents reported that their child felt uncomfortable with the length of the injection sequence and were worried that their own vaccination experience would last as long as it did in the game. In addition, parents expressed concern over the “suction noise” sound effect that was paired with the injection and described it as “off-putting.” Parents also reported that their child had difficulties using their finger to move the needle into place to start the injection sequence. Furthermore, 10 (29%) out of 35 children’s engagement tapered off over the course of the study gameplay period “since it was this same exact thing every day.” Other barriers experienced by 4 (11%) out of 35 children included thinking the game was “too loud” or there being technical difficulties where the sound did not play at all.

### Impact of the Game

#### Quantitative Results

Quantitative and qualitative findings demonstrated that support for the game positively affected children’s fears and physical reactions to needles. On the basis of retrospective reports in the exit survey, Wilcoxon signed rank tests revealed that parents rated their child’s fear as significantly lower during needle-related activities after participating in the study (median 3; mean 3.09, SD 1.17) compared with their retrospective report of their child’s fear in previous years before participating in the study (median 5; mean 4.37, SD 0.81; *z*=−4.638; *P*<.001). Similarly, parents rated their children’s distress (eg, crying, screaming, and kicking) during needle-related activities as significantly lower after participating in the study (median 3; mean 2.97, SD 1.42) compared with their retrospective report of their children’s fear in previous years before participating in the study (median 5; mean 4.37, SD 0.88; *z*=−4.313; *P*<.001). However, there was no significant difference between the initial fear scale ratings (median 1; mean 0.97, SD 1.18) and the final fear scale ratings (median 0; mean 0.69, SD 1.13; *z*=−1.624; *P*=.10).

#### Qualitative Results

Qualitative results further supported the positive impact the game had on children’s experience with needles, with 26 (74%) out of 35 children experiencing a reduction in fear of needles (21/35, 60%) or physical reactions (17/35, 49%) at the time of the medical appointment involving needles (as reported by their parents). However, the extent of the reduction varied. Some parents described a drastic improvement in their children’s fear and physical reactions after playing Dr. Zoo compared with their previous needle appointment before participating in the study:

Last year during [child’s] checkup for her vaccines, she hid under the table and was crying. We also had to wrestle her out of her clothes, hold her legs down. [This time], she got up on the table, she got her arm out, and then she leaned into me... and she said, ‘I’m ready, Mama... It’s, it’s just gonna be a pinch.’ And she just took a deep breath, and it was done. And she looked at me and she said, ‘That was easy.’ And I was shocked. But it was amazing. And I think a big part of it was playing the game.

For other children, they showed little fear before the appointment, but were “still screaming and kicking” when the time came for the actual injection. In contrast, some parents felt that their child was “still fretting a decent amount” about their needle appointment, but there was “no crying” this time around.

Parents of 21 (60%) out of 35 children also appreciated that the game provided an opportunity to mentally prepare their child for the vaccine appointment, making their child “less nervous when it comes to [the appointment] because it gives them time to sit down and talk about it every single night leading up to it.” However, 1 parent described that she disliked when her son “repeatedly” asked why he was “playing this game and if that meant that he was going to get a shot himself.” Furthermore, having the game opened up the conversation with their children about the purpose and health impact of vaccines, as reported by the parents of 21 (60%) out of 35 children. One parent recounted what she would say to her child after having them play the game:

There are times when we need it, and they help us. Just like our dog gets shots. They make us healthy. Sometimes if we’re sick, we need them. And needles are not bad, they are just to help us.

Nonetheless, the parents of 10 (29%) out of 35 children felt that the game had no impact on either their child’s fear of needles or their physical reactions to needles. Furthermore, parents of only 2 (6%) out of 35 children in the study felt that the game potentially made their child’s fear surrounding needles worse because the “anticipation” of knowing that their needle appointment was around the corner made them more anxious.

### Suggestions for Future Development

Parents of 25 (71%) out of 35 children gave suggestions to add features to the game to be more realistic of their child’s actual needle experience. For example, parents suggested having the child place a bandage on the animal after the injection sequence to make the animal feel better, because their children felt a level of comfort after receiving a bandage after their vaccination. In accordance with some participants’ discomfort with the length and sound of the injection sequence, parents suggested reducing the duration of the injection sequence and changing the sound to a short click or having no sound at all. Another suggestion was to have the animals show some apprehension surrounding the vaccination but ultimately overcome their fear. Other suggestions included adding different medical procedures, such as blood draws and being able to place the needle in different areas of the body other than the arm. Parents of 18 (51%) out of 35 children also suggested including new animal chapters to “maintain the engagement level” throughout the gameplay period. Finally, parents of 11 (31%) out of 35 children suggested some technical improvements to the game, such as adding instructions on how to move the screen around using the arrows or being more explicit in how to interact with the animals in each chapter.

## Discussion

### Principal Findings

Overall, Dr. Zoo demonstrated strong acceptability, ease of use, and potential preliminary effectiveness in this pilot feasibility study. Parent participants provided insightful feedback on the facilitators of and barriers to use, which will be helpful in the future development of the game.

Both quantitative and qualitative results showed that participants’ satisfaction with the game was high, with many children being excited to play the game each day. In addition, parents reported that the game was easy for their children to use, although younger children had a more difficult time using the game. In addition, the chapters that required greater input from the player proved to be a barrier for some players. In particular, players had issues with both the Penny the penguin and Sami the orca chapters, which required greater game literacy to navigate 3D spaces.

Dr. Zoo also demonstrated potential preliminary effectiveness in decreasing needle anxiety, although concurrent and retrospective data appear to be conflicting. Exit surveys and interviews indicated overwhelmingly that parents found the game successful, suggesting perceived decreases in fear of needles and improvements in distress to needles during appointments. However, fear scale ratings completed by parents at the end of individual play sessions indicated no decrease in children’s anxiety. A possible explanation for these contradictory results could be that the fear scale rating questionnaires captured players’ fear of the game itself rather than their fear of actual needles. Another potential reason for this result could be that the fear scale ratings demonstrated a floor effect, which could have prevented the detection of statistical significance. Low fear scale ratings after play sessions indicated that players did not experience high levels of fear while playing Dr. Zoo throughout the gameplay period.

Parents used Dr. Zoo as an opportunity to educate their child about the importance of vaccines to their health and prepare them for their needle appointment. Doing so may have helped normalize the experience of receiving a vaccination, provided context for the vaccination experience, and encouraged the child to communicate their fears with their parents, all of which may have contributed to Dr. Zoo’s effectiveness, despite these experiences lying outside the gameplay itself. In contrast, being reminded of their needle appointment each day while playing the game had the opposite effect for 2 children, making them more nervous in anticipation of their appointment.

Dr. Zoo’s success is especially notable given its uniqueness, when compared with prior work, as a serious game that applies in-game exposure therapy techniques without augmented reality or VR. Limitations in our fear measurements made our primary analysis qualitative, which made it difficult to directly compare the effectiveness of our intervention to the body of literature with more rigorous and quantitative support for the effectiveness of VRET solutions [11]. Parent-reported fear of needles in exit interviews changed from a mean rating of 4.37 to 3.09, exhibiting a 29% reduction in fear over the course of our study. Garcia-Palacios et al [33], in their study with the largest effect size as summarized by Opriş et al [11], found that a VRET app reduced self-reported fear of spiders, from a spider fear questionnaire [34], from 97.42 on average to 57.42, or about a 41% reduction, which is superior to Dr. Zoo. Although burdened by similar limitations, we can roughly compare our results to the state-of-the-art interventions specific to needle anxiety in children. Compared with 2 VR distractions by Özalp Gerçeker et al [35], which reported approximately 25% fear reduction on average, Dr. Zoo appears comparable in effect while having the advantage of not changing procedures of the medical appointment itself.

The Dominic the dragon chapter was a standout favorite of the 4 chapters, despite it being the most limited of the chapters in terms of opportunities for interaction with meaningful gameplay consequences. It is noteworthy that the Dominic the dragon chapter is on the leftmost point in the chapter selection screen (refer to Figure 1) and therefore may have regularly been chosen as a player’s first interaction with the game, suggesting that players may have been biased toward it as a result of sequencing rather than particular content. However, it is perhaps more compelling that, at odds with the frustration found in the Penny the penguin chapter, Dominic the dragon’s chapter has comparatively little opportunity to frustrate the player with difficult verbs. In terms of how the software reacts to input, the player can only (1) rotate the game environment to better view Dominic and (2) speaking into their microphone could decrease the time it takes for Dominic to calm down from 30 seconds to a minimum of 15 seconds.

Neither of these inputs create opportunities for agency comparable to the direct manipulation of objects found in the other 3 chapters (ie, throwing a ball to make Max fetch, placing a fish to make Penny walk, or tapping on trash objects to make them disappear). However, the fiction of the interaction with Dominic proved very powerful. Exit interviews indicated that players took the opportunity to engage in an emotional mirroring dialogue with the character that fundamentally aligns with our proposed empathetic mechanism. Dr. Zoo bears some similarity to Lumi Nova [12], mentioned earlier in the *Related Works* section, in that it requires the player to help nonplayer characters through anxiety issues that may resemble the player’s issues. This intersection suggests that the narrative premise of the player acting in a supportive role toward others with anxiety is a therapeutic design feature worth further deployment and study.

### Limitations

While discussing this work, it is important to acknowledge its limitations. The children who participated in this study were overwhelmingly White and non-Hispanic. The parents of these children were overwhelmingly White, non-Hispanic, women participants, and of the highest income category (>US $100,000/y). Children in households with higher socioeconomic status have less weekly screentime on average (including specifically video gameplay) than children in households with lower socioeconomic status [36]. This means that our game had novelty with this particular audience that may not be generalizable to children of lower socioeconomic backgrounds. In addition, although this often differs by specific vaccine, high-income White mothers are a highly vaccine-hesitant demographic, being the most likely to refuse vaccines for their children [37]. Therefore, there may be a recruitment bias, where our sample is overrepresented by the even more specific demographic of high-income White mothers who are *not* vaccine hesitant, potentially inflating the acceptability of the game compared to the general population’s attitudes.

Given our interpretation of the fear scale questionnaires, we are left with little rigorous means to quantify how much the game improved anxiety over time. Our only measure of children’s needle anxiety comes from the FSSC-R needle phobia item assessed at baseline to determine eligibility and their parent’s retrospective assessment in exit surveys and interviews, which one may criticize as an improper proxy.

Because this was a pilot feasibility study, we did not have a control group to compare children’s fear of needles between those who played the game and those who did not. Although parents provided varied feedback on the game, it is possible that receiving compensation for participating in the study biased their feedback. In addition, variability in how much parents encouraged their child to play the game or whether they let their child know about the upcoming needle appointment was not systematically assessed or controlled. Therefore, parents’ impact on their child’s experience with the game could not be measured. Furthermore, the children’s previous experience with other games was not assessed and therefore could not be controlled for in the analyses.

### Future Work

Further iterations of Dr. Zoo will focus on integrating opportunities for emotional mirroring, as seen in the Dominic the dragon chapter, into other chapters. Similarly, rewrites of the game’s narrative and dialogue may be necessary to further facilitate the prospective empathetic mechanism. Exit interviews indicated a pattern of confusion particularly around the Penny the penguin chapter, which focuses on Penny trying to search for Becky the polar bear, despite them standing next to each other in the main menu screen. In addition, none of the characters have any facial animation. Especially considering that we believe the player’s empathy for these characters plays an important role in the effectiveness of this game as a treatment for needle anxiety, the narrative and animation of these characters should come under closer scrutiny in future iterations.

Future evaluation of Dr. Zoo should use more rigorous assessments of the game experience, such as the Game Experience Questionnaire [38], and the impact of the game on children’s fear and perception of needles. A randomized controlled trial comparing needle anxiety in children who underwent the intervention to those who did not would clarify Dr. Zoo’s effectiveness. In addition, standardized measures should be used both before and after playing the game to assess the reduction in fear more accurately. Furthermore, parents’ involvement and children’s previous experience with games should be assessed and controlled in future studies.

### Conclusions

Dr. Zoo is one of the first exposure-based mobile games designed to reduce needle anxiety in young children aged 3 to 6 years. The results of this pilot feasibility study demonstrated that Dr. Zoo had high ease of use, acceptability, and potential preliminary effectiveness. Qualitative findings provided context for the quantitative findings and revealed that children had little difficulty playing the game and were excited to play the game each day. Furthermore, parents found the game to positively impact their child’s fear of and distress toward needles. Taken together, these results suggest that an evidence-based serious mobile game can be an acceptable and potentially effective intervention for changing young children’s fear and perceptions of needles. Exit interviews with parent participants revealed helpful suggestions for future iterations of the game, such as opportunities for children to display emotional mirroring with the animals and changes in ease of use. Leveraging digital interventions may be a potential solution to needle anxiety as a public health concern. As more mobile games are being developed to combat anxiety, it is imperative to integrate both evidence-based components and user input to achieve the highest impact.

## Appendix

Multimedia Appendix 1 Logistic regression analyses of children’s age predicting various barriers to use factors.

## Abbreviations

CBT: cognitive behavioral therapy
FSSC-R: Fear Survey Schedule for Children–Revised
VR: virtual reality
VRET: virtual reality exposure therapy

## Data Availability

Data are available from the corresponding author upon reasonable request.
