# Supplementary material for: An Exposure-Based Video Game (Dr. Zoo) to Reduce Needle Phobia in Children Aged 3 to 6 Years: Development and Mixed Methods Pilot Study
Source: JMIR Serious Games. 2023 Oct 16;11:e42025. doi: 10.2196/42025 (PMC10624234; doi:10.2196/42025)
Supplement: Multimedia Appendix 1 [file games_v11i1e42025_app1.docx]

| Table S1. Logistic Regression Analyses of Children’s Age Predicting Various Barrers to Use Factors | | | |
| --- | --- | --- | --- |
| **Dependent Variables** | **OR** | **CI** | ***p*** |
| Difficulty navigating space | .632 | .336 – 1.190 | .155 |
| Injection sequence | 1.496 | .789 – 2.835 | .217 |
| Loss of interest | .766 | .389 – 1.509 | .441 |
| Other barrier | 1.069 | .415 – 2.752 | .891 |

Note. CI = 95% Confidence Interval. OR = Odds Ratio.
